# Context-Dependent Roles of RNA Modifications in Stress Responses and Diseases

**DOI:** 10.3390/ijms22041949

**Published:** 2021-02-16

**Authors:** Emma Wilkinson, Yan-Hong Cui, Yu-Ying He

**Affiliations:** Department of Medicine, Section of Dermatology, University of Chicago, Chicago, IL 60637, USA; ewilkinson@uchicago.edu (E.W.); cuiyh88@medicine.bsd.uchicago.edu (Y.-H.C.)

**Keywords:** RNA modifications, m^6^A, cellular stress, disease

## Abstract

RNA modifications are diverse post-transcriptional modifications that regulate RNA metabolism and gene expression. RNA modifications, and the writers, erasers, and readers that catalyze these modifications, serve as important signaling machineries in cellular stress responses and disease pathogenesis. In response to stress, RNA modifications are mobilized to activate or inhibit the signaling pathways that combat stresses, including oxidative stress, hypoxia, therapeutic stress, metabolic stress, heat shock, DNA damage, and ER stress. The role of RNA modifications in response to these cellular stressors is context- and cell-type-dependent. Due to their pervasive roles in cell biology, RNA modifications have been implicated in the pathogenesis of different diseases, including cancer, neurologic and developmental disorders and diseases, and metabolic diseases. In this review, we aim to summarize the roles of RNA modifications in molecular and cellular stress responses and diseases.

## 1. Introduction

RNA modifications are covalent chemical modifications of RNA molecules. To date, over 100 chemical modifications of RNA species have been identified [1,2]. The regulation and function of RNA modifications have recently emerged as pivotal mechanisms that regulate a wide range of biological and pathological processes, giving rise to the field known as epitranscriptomics. 

**Figure 1:**
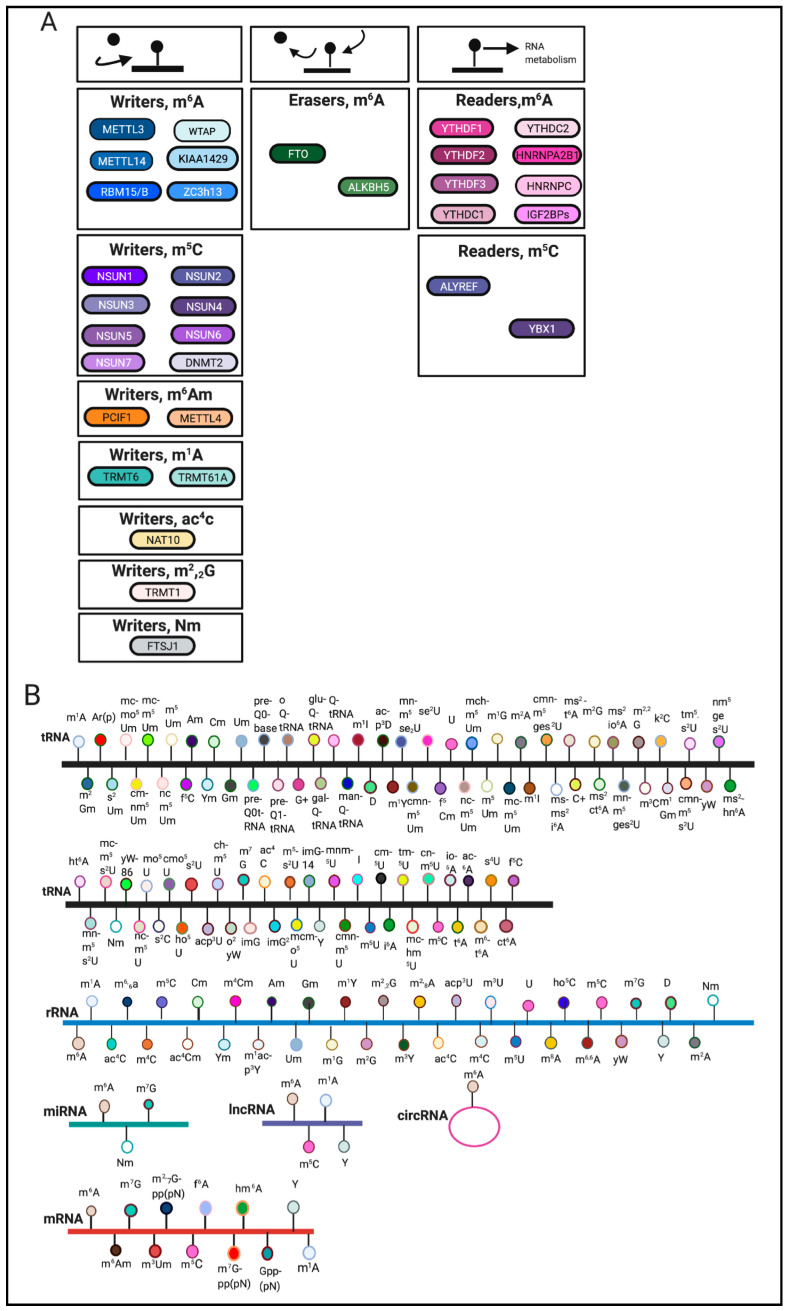
Overview of RNA modifications. (**A**). Writers, erasers and readers involved in catalyzing various RNA modifications. (**B**). Noted are RNA modifications that have been identified on tRNA, rRNA, mRNA, miRNA, lncRNA, circRNA [1,2,6,7,8,9,10,11]. The schematic was created using BioRender.

RNA modifications are regulated through the coordination of ‘writers’, ‘erasers’ and ‘readers’, which deposit, remove, and recognize RNA modifications, respectively (Figure 1A). These enzymes represent key elements in patterning the epitranscriptomic landscape. 

RNA modifications can occur on various RNA species including mRNA, tRNA, rRNA and other non-coding RNAs [3] (Figure 1B). Among these RNA species, transfer RNAs (tRNAs) contain the most RNA modifications [4]. One of the most abundant modifications on tRNA and rRNA is 5-methylcytosine (m^5^C) [5]. In comparison, mRNA modifications were more difficult to identify and characterize due to their low-abundance. The advent of sophisticated sequencing technologies and methods has generated a renewed interest in mRNA modifications. 

The most abundant internal mRNA modification is N^6^-methyladenosine (m^6^A) [2]. Other mRNA modifications are reviewed elsewhere [2,3,12]. m^6^A writers include methyltransferase-like family members 3,14 (METTL3, METTL14), Wilms tumor 1-associated protein (WTAP), KIAA1429, RBM15/RBM15B, and Zc3h13 [2,13,14]. m^6^A erasers include fat mass and obesity associated (FTO) and Alkb homologue 5 (ALKBH5) [2]. Furthermore, m^6^A readers include YTH m^6^A-binding protein 1,2 and 3 (YTHDF1, YTHDF2, YTHDF3), Heterogeneous nuclear ribonucleoprotein A2/B1 and C (HNRNPA2B1, HNRNPC), YTH domain-containing 1 and 2 (YTHDC1, YTHDC2), and IGF2BPs [2,9,15]. m^6^A writers, erasers and readers are reviewed elsewhere [2,16]. In this review, we aim to summarize the role of RNA modifications in cellular stress response pathways and within various diseases. m^6^A remains the best-studied RNA modification and will therefore be the main focus of this review.

## 2. RNA Modifications in Stress Responses

### 2.1. Oxidative Stress

Both m^6^A and m^5^C pathways play important roles in regulating the cellular response to oxidative stress, a condition caused by disrupted redox homeostasis, including the generation of reactive oxygen species (ROS). 

#### 2.1.1. Writers

**Figure 2:**
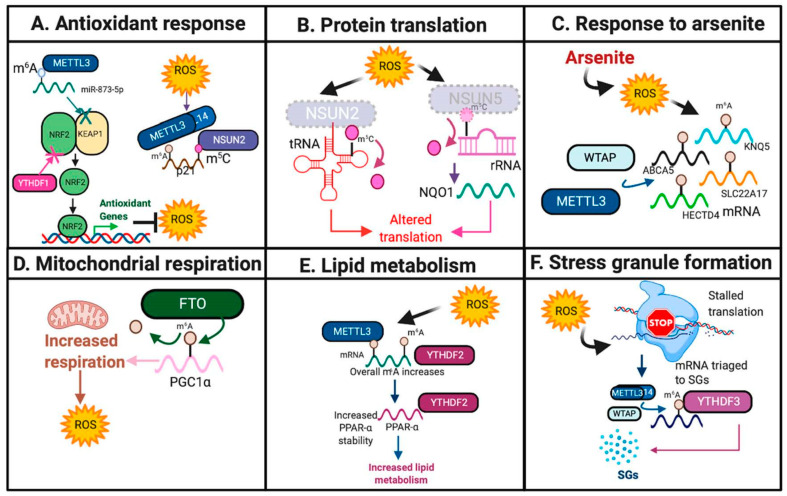
RNA modifications in response to oxidative stress. Highlighted are the diverse pathways and mechanisms by which RNA modifications respond to oxidative stress. Featured pathways include: (**A**). Antioxidant response. (**B**). Protein translation. (**C**). Response to arsenite. (**D**). Mitochondrial respiration. (**E**). Lipid metabolism. (**F**). Stress granule formation. The schematic was created using BioRender.

**Figure 3:**
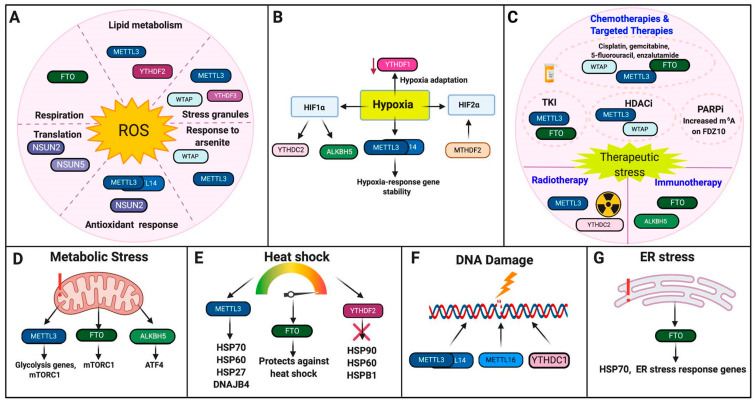
RNA Modifications in cellular stress responses. Highlighted are the roles of RNA modifications in response to cellular stresses. (**A**). Oxidative Stress. (**B**). Hypoxia. (**C**). Therapeutic Stress. (**D**). Metabolic Stress. (**E**). Heat Shock. (**F**). DNA damage. (**G**). ER Stress. The schematic was made using BioRender.

Previous studies have suggested that METTL3 may serve a protective role against oxidative stress. In mouse renal tubular epithelial cells, METTL3-mediated m^6^A deposition onto miR-873-5P and promoted its recognition and maturation by DGCR8, an miRNA processing complex, leading to inhibition of KEAP1 and activation of the NRF2 antioxidant pathway in response to colistin-induced oxidative stress [17,18] (Figure 2A and Figure 3A, Table 1).

Furthermore, m^5^C writers have also been implicated in the oxidative stress response. Using colon cancer cell lines and HeLa cells, Li and colleagues identified that NSUN2 catalyzes the deposition of m^5^C, and METTL3/METTL14 catalyze the deposition of m^6^A, in the 3′ UTR of *p21*, which has been previously found to up-regulate *NRF2* in response to oxidative stress and induce cellular senescence [20,60]. These results therefore suggest that these methyltransferases synergize to increase *p21* expression in response to oxidative stress [20]. Furthermore, *nsun2* expression was decreased upon oxidative stress in mouse keratinocytes, leading to site-specific reductions in m^5^C on tRNAs, stalling protein translation, and forcing the cell to enter into a catabolic state [23]. These results suggest that loss of NSUN2 may be necessary to induce the cellular stress response [23] (Figure 2B and Figure 3A, Table 1).

In human keratinocytes, ROS was induced by arsenite, an environmental carcinogen, resulting in increased expression of *WTAP* and *METTL14* and overall m^6^A levels [22,61]. Specific m^6^A increases were also identified on cell surface receptor *SLC22A17*, potassium channel *KCNQ5*, ATP binding cassette subfamily A member 5 (*ABCA5*) and HECT domain E3 Ubiquitin Protein Ligase 4 (*HECTD4*), leading to decreased mRNA expression of these genes [22] (Figure 2C and Figure 3A, Table 1). Further studies are needed to elucidate the functional significance of these targets in this context. 

Additionally, in glioma, epigenetic loss of NSUN5 resulted in hypomethylation of 28S rRNA at position C3782, leading to decreased overall protein translation in response to oxidative stress, but increased translation of proteins that promote survival and adaptation to oxidative stress, including antioxidant NQO1 [24] (Figure 2B and Figure 3A, Table 1).

#### 2.1.2. Erasers

m^6^A demethylases have also been implicated in the oxidative stress response. Zhuang and colleagues identified that in clear cell renal carcinoma, FTO induces oxidative stress through m^6^A demethylation at the 3′UTR of *PGC1α*, a major regulator of mitochondrial metabolism, which resulted in increased *PGC1α* mRNA stability and translation and increased ROS production [27] (Figure 2D and Figure 3A, Table 1). Furthermore, overexpression of *FTO* in hepatocytes and myotubes resulted in increased lipogenesis and mitochondrial dysfunction, which, in turn, increased ROS levels and induced oxidative stress [25,26]. 

#### 2.1.3. Readers

m^6^A readers serve diverse roles in response to oxidative stress. Loss of clock protein BMAL1 increased ROS production in human HepG2 and mouse Hepa1-6 cells, which resulted in specific METTL3-mediated m^6^A increases on the nuclear receptor peroxisome proliferator-activator α (*PPAR*α) locus, increased *YTHDF2* expression, which mediates *PPAR*α stability, and increased lipid metabolism [21] (Figure 2E and Figure 3A, Table 1). However, YTHDF1 and YTHDF2 may serve context-dependent functions in mediating oxidative stress. YTHDF1 may serve as a negative regulator of the KEAP1-NRF2 antioxidant pathway as *YTHDF1* knockdown in human bronchial epithelium cells (BEAS-2B) increased *NRF2* expression and antioxidant production [28]. 

Oxidative stress also induced METTL3/METTL14/WTAP-mediated m^6^A deposition on 5′UTR of stress granules (SGs), which are assemblies of mRNA that are stalled within translation initiation, and form in response to stress [19,62,63]. YTHDF3 has been shown to mediate the triage of mRNAs into SGs in response to oxidative stress in HEK293 and U2OS osteosarcoma cells [19] (Figure 2F and Figure 3A, Table 1).

### 2.2. Hypoxia

While the genetic mechanisms that mediate the hypoxia response have been a subject of study for many years, post-transcriptional regulation of the hypoxia response is not as well-elucidated. Emerging evidence suggests that m^6^A RNA methylation has an active role in cellular response to hypoxia (Figure 3B, Table 1).

### 2.2.1. Writers

METTL3/METTL14 activity and m^6^A deposition can be regulated by hypoxia. Overall m^6^A levels were increased under hypoxia in HEK293T cells, including specific m^6^A increases on hypoxia-responsive genes *GLUT1* and c-*MYC* [29]. Additionally, METTL3/14 promoted the mRNA stability of hypoxia-response genes, suggesting that m^6^A is involved in post-transcriptional stabilization of hypoxia-induced mRNAs [29]. Furthermore, *METTL3* was identified as a hypoxia-responsive gene in both endothelial cells and adipocyte stem cells upon differentiation into vascular smooth muscle [30,31]. 

However, other groups have found that m^6^A levels increased in response to hypoxia irrespective of METTL3/METTL14 or ALKBH5 activity in transformed human mammary epithelial cells, suggesting that there may be other novel hypoxia-induced mechanisms that regulate m^6^A levels [64]. Correspondingly, methylenetetrahydrofolate dehydrogenase 2 (MTHDF2), an enzyme that is important in one-carbon metabolism, is believed to regulate m^6^A deposition on *HIF-2α* in renal cell carcinoma (RCC), which leads to increased *HIF-2α* translation, metabolic reprogramming, and tumor progression [65,66].

### 2.2.2. Erasers

Thalhammer and colleagues identified that the *ALKBH5* promoter contains two putative binding sites for *HIF-1α* and was up-regulated in response to hypoxia across several different cancer cell lines, including U2OS, breast cancer cell line MCF7, and neuroblastoma cell line IMR32 [32]. However, the hypoxia-induced changes in m^6^A demethylation and patterning across these cell lines requires further study. Additionally, ALKBH5 and ZNF217 were found to regulate a breast cancer stem cell (BCSC) phenotype in hypoxia, resulting in decreased m^6^A methylation of *NANOG* and *KLF4* and increased expression of these pluripotency factors [33,34].

### 2.2.3. Readers

The role of m^6^A readers in response to hypoxia requires further study. Developmentally, lower *YTHDF1* expression was seen in mammals that live at low oxygen altitudes, suggesting that loss of YTHDF1 may serve as an evolutionary adaptation to hypoxia [28]. Furthermore, YTHDC2 was found to promote *HIF-1α* mRNA translation in colon cancer [35]. 

### 2.3. Therapeutic Stress

Resistance to therapies remains a pressing issue for many types of cancer. Writers, erasers, and readers for m^6^A RNA methylation are shown to play important roles in response to therapeutic agents, including targeted therapies, immunotherapies, and conventional therapies (Figure 3C, Table 1). Further elucidating the epigenetic mechanisms that mediate chemotherapy resistance remains an active area of research. 

#### Chemotherapies and Targeted Therapies

*Cisplatin, gemcitabine, 5-fluorouracil, enzalutamide. METTL3* expression may induce resistance to chemotherapy as *METTL3* knockdown increased sensitivity to gemcitabine, 5-fluorouracil (5-FU) and cisplatin in pancreatic cancer, potentially through activation of MAPK signaling [38]. In glioma, METTL3 may also promote temozolomide resistance through m^6^A-mediated stabilization of *SOX2*, which mediates glioma stem cell formation, as *METTL3* knockdown increased temozolomide sensitivity in this context [39]. Additionally, in AML, *WTAP* knockdown increased sensitivity to etoposide, suggesting that WTAP may mediate etoposide resistance [40]. Furthermore, FTO increased resistance to cisplatin in cervical squamous cell carcinoma (CSCC) through m^6^A demethylation of the *β-CATENIN* transcript, leading to increased *β-CATENIN* mRNA and protein expression [45]. FTO also contributed to enzalutamide resistance in castration-resistant prostate cancer by mediating alterations within androgen receptor-regulated enhancer RNAs (AR-eRNAs) [44]. Therefore, whether m^6^A methylation or demethylation mediates chemotherapy resistance is cancer-cell type and context dependent. 

*Kinase Inhibitors*. Yan and colleagues reported increased FTO-mediated m^6^A demethylation increased resistance to tyrosine kinase inhibitors (TKIs) through enhanced mRNA stability of anti-apoptotic genes and pro-proliferation genes, such as *BCL-2*, in leukemia [42]. In liver cancer, decreased *METTL3* expression increased resistance to sorafenib through decreased mRNA stability of *FOXO3*, a negative regulator for autophagy, leading to the activation of autophagy-mediated therapeutic resistance [37]. 

*Histone Deacetylase (HDAC) Inhibitors.* In non-small cell lung cancer (NSCLC), chidamide, an HDAC inhibitor, inhibited *c-MET* expression by decreasing the m^6^A methylation of *c-MET*, potentially through targeting WTAP and METTL3 [36]. Additionally, chidamide-mediated decreases in m^6^A and *c-MET* expression also increased sensitivity to critzotinib [36].

*PARP Inhibitors (PARPi).* Increased m^6^A deposition on *FZD10* mRNA resulted in increased *FZD10* stability, which mediated resistance to PARPi through activation of the WNT/*β-CATENIN* pathway in BRCA-mutant epithelial ovarian cancer cells [67]. 

***Immunotherapies***. In melanoma, FTO promoted the expression of *PDCD1*, which expresses the PD-1 protein, as well as *CXCR4* and *SOX10*, promoting melanoma tumorigenesis and resistance to immunotherapy [43]. Decreased *FTO* expression therefore increased response to the PD-1 blockade immunotherapy as well as IFNy-induced tumor cell killing [43]. *ALKBH5* knockdown also increased PD-1 therapy efficiency in both melanoma and colorectal cancer models by decreasing populations of immunosuppressive Tregs and myeloid-derived suppressor cells (MDSCs) [41]. The role of m^6^A in mediating the immune response is complex and remains an active area of study.

***Ionizing Radiation***. *METTL3* expression promoted radioresistance in glioma by promoting glioblastoma stem cell maintenance through m^6^A-mediated stabilization of *SOX2*, and subsequent *METTL3* knockdown resulted in increased radiosensitivity [39]. In addition to promoting cisplatin resistance, FTO also contributed to radiation resistance in CSCC through up-regulation of β−CATENIN [45]. YTHDC2 contributed to radioresistance in nasopharyngeal carcinoma (NPC) cells by binding to *IGF1R* mRNA and promoting the translation of the *IGF1R* transcript, leading to downstream activation of the IGF1R-AKT/S6 signaling axis [46]. 

### 2.4. Metabolic Stress

Multiple writers, erasers, and readers for m^6^A RNA methylation are shown to play important roles in response to metabolic stress, which can be induced through nutrient stress, excessive energy production, or within tumorigenesis (Figure 3D, Table 1).

### 2.4.1. Writers

METTL3 may serve pivotal roles in glycolysis and metabolism and subsequent loss of METTL3 may therefore promote metabolic stress. In hepatocellular carcinoma (HCC), *METTL3* expression positively correlated with glycolysis genes *SLC2A1, HK2*, *PFKM* and P*KM* [47]. Knockdown of *METTL3* decreased mTORC1 activity, a major regulator of cellular metabolism, and also sensitized HCC cells to 2-deoxyglucose (2-DG), a glycolysis inhibitor, promoting glycolytic stress and the subsequent induction of cell death [47,68].

### 2.4.2. Erasers

ATF4 is a master regulator of amino acid metabolism [69]. Upon amino acid starvation and subsequent induction of nutrient stress in mouse embryonic fibroblasts (MEFs), *alkbh5* was recruited to *atf4* mRNA and promoted *atf4* translation, suggesting that m^6^A regulation at the *atf4* transcript contributes to the cellular stress response in response to nutrient stress [48]. 

Additionally, FTO was found to activate the mTORC1 pathway in MEFs as *fto* knockdown decreased mTORC1 activation and increased autophagy [49]. Furthermore, *fto* was down-regulated upon amino acid starvation, potentially as a means to regulate mTORC1 activation upon amino acid stress [49]. However, in melanoma, *FTO* was up-regulated in response to metabolic stress and promoted adaptation to metabolic stress, therefore reflecting the context-dependent roles of m^6^A in response to metabolic stress [43].

### 2.5. Heat Shock

The best-studied heat shock response involves the translation and regulation of heat shock proteins (HSPs), which function to mediate adaptation to heat stress.

Multiple writers, erasers, and readers for m^6^A RNA modifications have been shown to play important roles in regulating the expression of HSPs and within the heat shock response (Figure 3E, Table 1).

### 2.5.1. Writers

*METTL3* was decreased in HepG2 cells upon heat shock [52]. METTL3 may also target *HSP70, HSP60*, and *HSP27* downstream, as *METTL3* knockdown decreased the mRNA expression of these genes [52]. m^6^A-mediated regulation of HSPs is further corroborated by the identification of m^6^A sites on HSPs [50]. Other studies have also identified increased METTL3-mediated m^6^A deposition on the 5′UTR of *HSP70* post-heat shock, which is hypothesized to regulate cap-independent translation in response to heat stress [53]. Similarly, m^6^A increases were also identified in the 5′UTR of D*NAJB4* (*HSP40* Homolog) upon heat shock, but it is unclear whether METTL3 alone mediates this m^6^A increase [51]. These studies therefore highlight the context-dependent regulation of HSPs mediated by m^6^A.

### 2.5.2. Erasers

The role of m^6^A erasers in response to heat shock is not well-established. An overall decrease of m^6^A and increase in *fto* was noted in response to a mild heat challenge in the hypothalamus of male Cobb chicks, suggesting that FTO serves a protective role against heat stress [54]. However, during a harsher heat challenge, m^6^A levels increased, suggesting that m^6^A regulation may be temperature-dependent [54].

### 2.5.3. Readers

*YTHDF2* mRNA was increased upon heat shock, but also may serve as a negative regulator of *HSP90, HSP60*, and *HSPB1* as *YTHDF2* knockdown resulted in increased mRNA expression of these HSPs in HepG2 cells [52]. Zhou and colleagues also discovered that m^6^A is preferentially deposited on the 5′UTR of newly transcribed *HSP70* mRNA and is read by YTHDF2 upon heat shock, which may be functionally significant as previous reports have found that m^6^A deposition at the 5′UTR mediates cap-independent translation [55,70].

### 2.6. DNA Damage

m^6^A RNA methylation has been shown to facilitate DNA repair and DNA damage response under genotoxic stress (Figure 3F, Table 1).

### Writers

In response to UVC/UVA radiation, m^6^A and DNA Pol κ were rapidly recruited (2–4 min post-UV) to sites of DNA damage by METTL3/METTL14 [56]. METTL16, a U6 snRNA methyltransferase, was also recruited to DNA damage sites at a later time point (20–30 min post-UV) and was found to methylate small nuclear and nucleolar RNAs that were also recruited to DNA damage sites upon UV radiation [58]. m^6^A was only recruited to DNA damage lesions only in the presence of cyclobutane pyrimidine dimers (CPDs), which form in response to UV exposure [58]. Furthermore, in response to UV, m^6^A RNA modifications may utilize the nucleotide excision repair (NER) pathway rather than non-homologous end-joining (NHEJ), as knockout of two NHEJ-specific enzymes, *SUV391H/H2*, had no effect on m^6^A recruitment [58]. In response to double-stranded breaks (DSBs), METTL3 localized to DNA damage sites, depositing m^6^A on DNA-damage associated RNA, increasing accumulation of DNA-RNA hybrids, and recruiting YTHDC1 and DNA-damage associated proteins RAD51 and BRCA to initiate homologous recombination (HR) [57]. 

### 2.7. ER Stress

The role of RNA modifications in ER stress remains understudied in mammalian systems. Work in non-mammalian systems suggests that ER stress may influence or induce m^6^A deposition in response to viral infection and innate immune signaling [71]. The limited work in mammalian systems suggests that FTO stabilizes *HSP70* and other ER stress-associated genes in response to genotoxic stress in osteoblasts [59] (Figure 3G, Table 1). 

## 3. RNA Modifications in Diseases

### 3.1. Cancer

**Figure 4:**
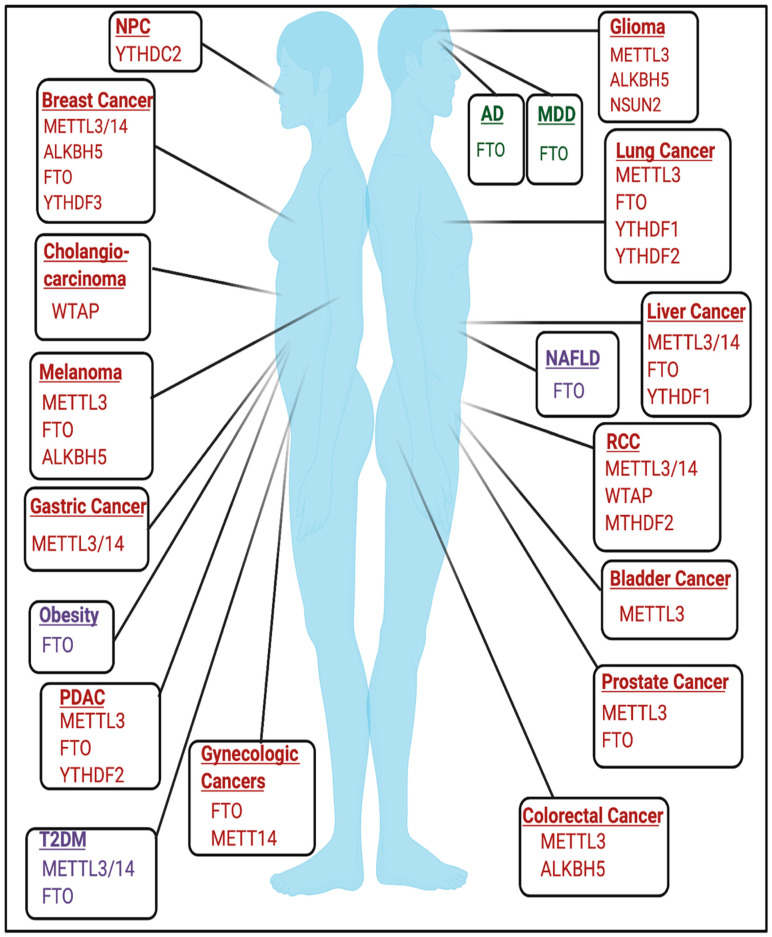
RNA Modifications in diseases. Highlighted are the regulators of RNA modifications that have established roles in regulating disease pathogenesis across genders as well as sex-specific diseases such as breast cancer, gynecologic cancers, and prostate cancer. Windows in red are modifiers implicated in cancer. Windows in purple are metabolic diseases. Windows in green are neurologic diseases. Not pictured are developmental disorders. The schematic was created using BioRender.

The role of RNA modifications in cancer is cell-type and context-dependent and has been reviewed extensively in various types of cancer [16,72,73,74,75,76,77,78,79,80,81] and are highlighted in Figure 4. Rare RNA modifications have been described in cancer type-specific contexts, such as glioblastoma, and are detailed in [82]. An active area of research seeks to elucidate the role of RNA modifications in non-coding RNAs and other RNA species in the context of cancer, which are reviewed elsewhere [83].

### 3.2. Developmental and Neurologic Disorders

The role of RNA modifications in the context of developmental and neurological disorders remains an active area of study. m^6^A has been previously found to play important roles in embryonic development and neurobiological functions [84,85]. The roles of m^6^A and other RNA modifications in mediating neurologic function are further discussed elsewhere [84,86,87,88,89].

The necessity of m^6^A in development is emphasized by early embryonic lethality in *mettl3* KO mice [90]. Conditional *mettl3* knockout in murine brains also resulted in severe developmental defects within the cerebrum and cortex and induced apoptosis in cerebella granule cells (CGCs) [91]. FTO may also be important in mediating development as expression of catalytically inactive mutant *FTO*(R316Q) resulted in severe growth defects [92]. Mutations in tRNA methyltransferases have been implicated in developmental disorders and are detailed in [93]. *NSUN2* mutations have been linked to microcephaly, intellectual disability, and Dubowitz Syndrome, which is characterized by growth and mental retardation [94,95,96]. Additionally, homozygous frameshift mutations in *TRMT1*, a writer for m^2^,_2_G, have been linked to intellectual disability [97]. Mutations and polymorphisms in *FTSJ1*, a writer for 2′O-methylribose, have also been linked to X-linked mental retardation [98,99,100,101,102]. Furthermore, targets of fragile X mental retardation protein (FMRP), a protein that is commonly mutated in Fragile X Syndrome, were enriched for m^6^A, and FMRP targets were targeted for degradation by YTHDF2 [103].

#### 3.2.1. Alzheimer’s Disease 

m^6^A increases and distinct m^6^A patterning were found in the cortex and hippocampus of APP/PS1 transgenic mice, which are used to model Alzheimer’s Disease (AD) [104]. Additionally, AD-associated SNPs that decreased *FTO* expression were identified in Caucasian and Caribbean Hispanic populations [105]. AD patients also showed changes in small RNA modifications, which are detailed in [89]. 

#### 3.2.2. Major Depressive Disorder

m^6^A and m^6^Am may be also implicated in major depressive disorder (MDD) as m^6^A and m^6^Am patterning were dysregulated in patients with MDD [106]. Conversely, the *FTO* variant rs9939609 was associated with a lower risk of developing MDD [107,108]. FTO may be also be involved in the development of anxiety, as *fto*^−/−^ mice show increased anxiety-like behavior and hyperactivation of the hypothalamic-pituitary-adrenal (HPA) axis [109]. 

### 3.3. Metabolic Disorders and Diseases

FTO is a major driver in contributing to the pathogenesis of several metabolic diseases and therefore serves as a therapeutic target in this context. 

#### 3.3.1. Obesity

One of the strongest predictors of obesity is believed to be SNP rs9939609 in *FTO* [110,111,112,113,114,115]. *FTO* SNPs rs17817449 and rs3751812 also increased obesity risk in north Indian and Pakistani populations, respectively [116,117]. *fto* overexpression in mice also resulted in a dose-dependent increase in body mass and increased food intake [118,119]. However, these studies are m^6^A-independent. Conversely, previous studies have identified that FTO-mediated m^6^A demethylation regulates mRNA splicing in adipocytes and genes involved in sterol metabolism, which therefore may provide a mechanism by which FTO promotes obesity at the molecular level [120]. The role of FTO in metabolism is further reviewed elsewhere [121]. Identifying the m^6^A-dependent and m^6^A-independent functions of FTO in mediating obesity remains an active area of research.

#### 3.3.2. Diabetes 

In addition to obesity, m^6^A sequencing of type-two diabetes mellitus (T2DM) patients revealed overall changes in m^6^A patterning and hypomethylation of mRNA transcripts involved in insulin biogenesis, secretion, and pancreatic β-cell biology [122,123]. *FTO* mRNA expression was also higher in some T2DM patients [124]. Additionally, *METTL3/METTL14* expression was decreased in β-cells of patients with T2DM, and METTL14 specifically may be essential for insulin secretion and β-cell survival [122,123,125]. However, the role of m^6^A in mediating T2DM may be tissue and context-dependent as *METTL3* and m^6^A levels were increased in liver tissue from T2DM patients [126]. 

#### 3.3.3. Non-Alcoholic Fatty Liver Disease 

Increased expression of *fto* was induced by a high-fat diet, resulting in increased lipogenesis and induction of non-alcoholic fatty liver disease (NAFLD), a disease that is commonly associated with obesity [26,127,128]. Identifying the m^6^A-dependent and m^6^A-independent functions of FTO in this context requires future study. 

## 4. Conclusions and Perspectives

Emerging evidence demonstrates the crucial role of RNA modifications in stress responses and diseases. These findings not only shine new lights on molecular and cellular responses to a wide range of stress conditions, but also may provide new opportunities in targeting the RNA modification pathways to modulate stress responses and thus may prevent and treat diseases. The field of epitranscriptomics will benefit from the following advances in the near future. First, elucidating the context and cell-type-specific role of RNA modifications will allow us to fully understand the precise and diverse roles and functions of these modifications in biology and diseases. Throughout this review, we have highlighted the role of RNA modifications in promoting or resisting certain cellular processes that are cell-type and context-dependent. For example, what mediates the changes in RNA modifications and their role on gene transcription as a cell transitions from homeostasis to stress, or within oncogenic transformation? Second, while many sequencing technologies have been developed to detect RNA modifications, there is a need to detect these modifications directly at the single base resolution across all regions within the gene body. Last, there is also a need to further characterize the specificity with which RNA modifications are deposited. Accordingly, identifying the molecular mechanisms that determine which RNAs, RNA species, RNA secondary and tertiary structures, and adenosines, or other nucleosides, are targeted for modification will be critical to determining the sequence specificity of these modifications. These important advances will further facilitate the understanding of the precise role for RNA modifications in physiology and pathology, and may reveal new opportunities for disease diagnosis, prognosis, prevention, or treatment. 

## Figures and Tables

**Table 1:** Summary of the role of RNA modifications in stress responses.

Stressor	Regulators	Response to Stresses	Cells or Organisms	References
**Oxidative Stress**	**METTL3/14**	Increases in response to ROS, activates p21/NRF2, deposits m^6^A onto SGs	mRTECs, HeLa, colon cancer cell lines, HepG2, Hepa1-6, HEK293, U2OS	[18,19,20,21]
	**METTL14**	Increased upon arsenite-induced ROS	Keratinocytes	[22]
	**WTAP**	Increased upon arsenite-induced ROS	Keratinocytes	[22]
	**NSUN2**	Alters protein synthesis, enhances *p21* translation	HeLa, colon cancer cell lines, keratinocytes,	[20,23]
	**NSUN5**	Alters protein synthesis	Glioma cell lines	[24]
	**FTO**	Promotes mitochondrial and lipogenesis-induced ROS	HEK293T/kidney cancer cell lines, myotubes, L02 cells	[25,26,27]
	**YTHDF1**	Negative regulator of NRF2	Tibetan mammals	[28]
	**YTHDF2**	Increases in response to ROS	HepG2, Hepa1-6	[21]
	**YTHDF3**	Selective SG-reader in response to oxidative stress	HEK293T, U2OS	[19]
**Hypoxia**	**METTL3**	Promotes stability of hypoxia-response genes	HEK293T	[29]
	**METTL3/14**	Hypoxia-response gene	ADSCs, HUVECs	[30,31]
	**ALKBH5**	Induced by HIF1α, promotes BCSC phenotype	U20S, MCF7 and other breast cancer cell lines, IMR32, HeLa	[32,33,34]
	**YTHDF1**	Promotes hypoxia adaptation	Tibetan mammals	[28]
	**YTHDC2**	Promotes *HIF1*α translation	HT29, HCT116, COS	[35]
**Therapeutic Stress**	**METTL3**	Sensitive to sorafenib but increased resistance to gemcitabine, 5-FU, cisplatin, temozolomide, and radiotherapy; targeted by chidamide	HEK293T, HCC, NSCLC, PDAC and AML cell lines, GBM tissues and GSCs	[36,37,38,39]
	**WTAP**	Promotes etoposide resistance and is targeted by chidamide	NSCLC and AML cell lines	[36,40]
	**ALKBH5**	Promotes resistance to anti-PD-1 therapy	Melanoma cell lines	[41]
	**FTO**	Promotes resistance to TKIs, cisplatin, enzalutamide, and anti-PD-1 therapies	Leukemia, melanoma, CSCC, protstate cancer, and ovarian cancer cell lines	[42,43,44,45]
	**YTHDC2**	Promotes radioresistance	NPC cell lines	[46]
**Metabolic Stress**	**METTL3**	Combats glycolytic stress	HCC cell lines	[47]
	**ALKBH5**	Promotes *ATF4* translation	MEF	[48]
	**FTO**	Promotes adaptation to metabolic stress and regulates mTORC1	Melanoma cell lines, MEF	[43,49]
**Heat Shock**	**METTL3**	Regulates m^6^A deposition onto *HSP70*, *HSP60*, *HSP27*, and *DNAJB4*	HepG2, M14, HeLa, HEK293T, male chickens,	[50,51,52,53]
	**FTO**	Serves a protective role	Male Cobb chicks	[54]
	**YTHDF2**	Negative regulator of *HSP90*, *HSP60* and *HSPB1*; *HSP70* reader	HepG2, HeLa, MEF	[52,55]
**DNA Damage**	**METTL3**	Recruited to DNA damage sites post-UV	HEK293T, U2OS, HeLa, A375, MEF, CAL-27	[56,57]
	**METTL16**	Recruited to DNA damage sites post-UV	MEF, HaCaT, U2OS, HeLa	[58]
	**YTHDC1**	Recruited to DNA-RNA hybrids, recruit HR proteins	HEK293T, U2OS, CAL-27	[57]
**ER Stress**	**FTO**	Induces ER stress pathways post-genotoxic damage	Osteoblasts	[59]

## Data Availability

Not applicable.
